# Nano-silver in drinking water and drinking water sources: stability and influences on disinfection by-product formation

**DOI:** 10.1007/s11356-014-2508-5

**Published:** 2014-01-24

**Authors:** A.-M. Tugulea, D. Bérubé, M. Giddings, F. Lemieux, J. Hnatiw, J. Priem, M.-L. Avramescu

**Affiliations:** Health Canada, Ottawa, Canada

**Keywords:** Nano-silver, Drinking water, Surface water, Chlorination, Disinfection by-products, Stability

## Abstract

Nano-silver is increasingly used in consumer products from washing machines and refrigerators to devices marketed for the disinfection of drinking water or recreational water. The nano-silver in these products may be released, ending up in surface water bodies which may be used as drinking water sources. Little information is available about the stability of the nano-silver in sources of drinking water, its fate during drinking water disinfection processes, and its interaction with disinfection agents and disinfection by-products (DBPs). This study aims to investigate the stability of nano-silver in drinking water sources and in the finished drinking water when chlorine and chloramines are used for disinfection and to observe changes in the composition of DBPs formed when nano-silver is present in the source water. A dispersion of nano-silver particles (10 nm; PVP-coated) was used to spike untreated Ottawa River water, treated Ottawa River water, organic-free water, and a groundwater at concentrations of 5 mg/L. The diluted dispersions were kept under stirred and non-stirred conditions for up to 9 months and analyzed weekly using UV absorption to assess the stability of the nano-silver particles. In a separate experiment, Ottawa River water containing nano-silver particles (at 0.1 and 1 mg/L concentration, respectively) was disinfected by adding sodium hypochlorite (a chlorinating agent) in sufficient amounts to maintain a free chlorine residual of approximately 0.4 mg/L after 24 h. The disinfected drinking water was then quenched with ascorbic acid and analyzed for 34 neutral DBPs (trihalomethanes, haloacetonitriles, haloacetaldehydes, 1,1 dichloro-2-propanone, 1,1,1 trichloro-2-propanone, chloropicrin, and cyanogen chloride). The results were compared to the profile of DBPs obtained under the same conditions in the absence of nano-silver and in the presence of an equivalent concentration of Ag^+^ ions (as AgNO_3_). The stability of the nano-silver dispersions in untreated Ottawa River water, with a dissolved organic carbon concentration of 6 mg/L, was significantly higher than the stability of the nano-silver dispersions in distilled, organic-free water. Nano-silver particles suspended in the groundwater agglomerated and were quickly and quantitatively removed from the solution. Our data confirm previous observations that natural dissolved organic matter stabilizes nano-silver particles, while the high-ionic strength of groundwater appears to favor their agglomeration and precipitation. As expected, nano-silver was not stable in Ottawa River water through the chlorination process, but survived for many days when added to the Ottawa River water after treatment with chlorine or chloramines. Stirring appeared to have minimal effect on nano-silver stability in untreated and treated Ottawa River water. The profile of DBPs formed in the presence of nAg differed significantly from the profile of DBPs formed in the absence of nAg only at the 1 mg/L nAg concentration. The differences observed consisted mainly in reduced formation of some brominated DBPs and a small increase in the formation of cyanogen chloride. The reduced formation of brominated congeners may be explained by the decrease in available bromide due to the presence of Ag^+^ ions. It should be noted that a concentration of 1 mg/L is significantly higher than nAg concentrations that would be expected to be present in surface waters, but these results could be significant for the disinfection of some wastewaters with comparably high nano-silver concentrations.

## Background, aim, and scope

Nano-silver (nAg) is increasingly used in consumer products from washing machines (Farkas et al. 2011) to textiles and façade coatings (Som et al. 2011), personal care products (EPA 2013), and “Magic Socks” (Benn and Westerhoff 2008). Hundreds of commercial products claim to contain or release nAg particles (Project on Emerging Nanotechnologies, 2013). In addition, of special interest to consumer drinking water safety are nano-silver-containing products and devices marketed for the disinfection of drinking water and recreational water (Dankovich and Gray 2011; WHO 2013).

A majority of the nano-silver released during the use of devices or after the disposal of consumer products ultimately finds its way, through the wastewater stream, to the wastewater treatment facility (Benn and Westerhoff 2008) and can eventually reach surface water bodies used as possible drinking water sources (Gottschalk et al. 2009, 2010; O’Brien and Cummins 2010). Concentrations of nano-silver particles expected in North American surface waters (at 2008 production levels) are in the nanogram per liter level (Gottschalk et al. 2009). The concentration of nano-silver reaching wastewater plants (WWPs) is expected to be three orders of magnitude higher than the concentration in drinking water sources (Gottschalk et al. 2009, 2010; O’Brien and Cummins 2010), although the speciation of silver in the WWP may strongly affect its potential solubility and bioavailability (Nowack 2010).

Natural waters contain natural nanomaterials: organic colloids usually described under the term dissolved organic matter (DOM) and measured as dissolved organic carbon (DOC). The main fractions that can be separated from natural water DOM are humic fractions, humic acid and fulvic acid, as well as a hydrophilic fraction (Abbt-Braun et al.; 2004; Ma et al. 2001). Typical DOC concentrations in Canadian drinking water sources are in the range of 0.5–12 mg/L (Health Canada unpublished data). DOM structure and surface properties are influenced by water pH, ionic strength, and metal ion concentrations (Ghabbour and Davies 2001).

It has previously been demonstrated that DOM interacts not only with nano-silver (Dubasa and Pimpanb 2008; Gilliland 2011) but also with Ag+ ions (Chen et al. 2012). The stability of DOM colloids is explained by the mutual rejection of the negative charges existing on the surfaces of their particles, a mechanism similar to the one for stabilizing nano-silver products using capping agents like polyvinylpyrrolidone (PVP) and citrate. In fact, humic fractions have been used to cap and stabilize nano-silver particles during their synthesis (Dubasa and Pimpanb 2008). This suggests that DOM will interact with and stabilize nano-silver particles. The stabilization effect appears to be dependent on the nature of the DOM (Adegboyega et al. 2013). A number of studies have confirmed this hypothesis in model (Gilliland 2011) and natural (Akaighe et al. 2011) water systems containing DOM colloids.

Also, during water disinfection, oxidizing agents (chlorine, chloramine, ozone, and UV) used to destroy infectious agents react with DOM and form disinfection by-products (DBPs). To date, over 500 DBPs have been identified in finished drinking water. The speciation and amounts of DBP produced are a function of the oxidizing agent used and reaction conditions (Richardson 2005). The large surface area of silver nanoparticles could promote surface processes (e.g., adsorption and catalysis) (Tiede et al. 2008). Some nAg materials have demonstrated catalytic properties not observed in the bulk form. Silver nanoparticles and silver nanocomposites have been used to catalyze many chemical reactions (Liu and Zhao 2009; Hamal and Klabunde 2007).

Little information is available about the stability of nano-silver materials in sources of drinking water, their fate during drinking water disinfection processes, and their interaction with disinfection agents and DBPs. However, risk assessments for nano-silver-containing materials should be supported by detailed information on the stability of nano-silver particles in natural waters and finished drinking water. We need to know the lifespan of particular types of nano-silver particles in the target waters (hours, days, and months?) and the role that certain water characteristics (e.g., DOM concentration, ionic strength, hardness, calcium, magnesium, and sodium concentrations) play in determining this lifespan. We also need to know how nano-silver particles react during oxidative water disinfection processes: do some of the nanoparticles survive the water disinfection process? Do they participate in any way in the disinfection reactions? Do they play a catalytic role and do they have a qualitative and/or quantitative influence on DBP formation? These are questions that our study seeks to answer using the limited model of one nano-silver product: the 10 nm, PVP-coated, monodisperse nano-silver. This study aims to investigate the stability of the 10-nm, PVP-coated nano-silver particles in different drinking water sources and in the finished drinking water when chlorine or chloramines are used for disinfection and to observe potential changes in the composition of DBPs formed when nAg is present in the source water during the disinfection process.

## Materials and methods

### Samples

The nano-silver product used as a model was the Bio pure, 10 nm, PVP-coated nano-silver from NanoComposix, San Diego, CA. This material will be referred to as “nano-silver.” A 1:10 dispersion of nano-silver in organic-free water was used to spike untreated Ottawa River water (ORW), treated Ottawa River water (OTW), organic-free distilled water (DW), and a commercial bottled ground water (GW) at concentrations of 5 and 1 mg/L for stability studies.

Solutions of Standard Humic Materials procured from the International Humic Substances Society (IHSS; http://www.humicsubstances.org) were also spiked with nano-silver at 5 mg/L concentrations and used to model the influence of various dissolved organic matter fractions on nanoparticle stability.

DW was an organic-free water produced in our laboratory by distillation of deionized water (from the reverse osmosis Milli-Q system) using KMnO_4_ acidified with sulfuric acid to destroy the dissolved organic matter prior to distillation (see Table 1 for selected characteristics of the water samples used).

**Table 1:** Selected water characteristics

	DOC (mg/L)	pH	Ca^2+^ (μg/L)	Mg^2+^ (μg/L)	Na^+^ (μg/L)
ORW	6.4	7.6	12,000	3,100	3,900
OTW	2.6	8.3	12,000	3,000	21,000
GW	0.4	7.6	40,000	9,300	3,500
DW	NA	4.7	NA	NA	NA
SRFA6	3.2	4.2	NA	NA	NA

GW was a commercial brand of bottled water (Labrador water from AquaTerra Corporation, Mississauga, ON) known to originate from a groundwater source. According to its manufacturer, the GW was disinfected by ozonation. The GW had a low dissolved organic matter content. ORW was the raw water used by the Britannia Drinking Water Plant in Ottawa, Ontario, collected from the Britannia Drinking water plant during the winter of 2012–2013. It is river water, with moderately high DOC content and moderate ionic strength. OTW was collected from the Britannia Drinking Water Plant in Ottawa, Ontario in the winter of 2012–2013. The disinfection process uses chlorine, then chloramine.

Swannee river fulvic acid standard solution (SRFA6) is a 6-mg/L solution in DW prepared in our lab. The Swannee River Fulvic Acid Standard from IHSS was dissolved in DW and stirred for at least 48 h before being used in experiments. SRFA06 is a 0.6-mg/L solution obtained by diluting the SRFA6 with DW.

The sodium hypochlorite solution was prepared from laboratory grade (5.65–6 %) sodium hypochlorite (Fisher Scientific, NJ) by dilution with DW. A solution of silver nitrate (Sigma-Aldrich Canada Co., Oakville, ON) with a concentration equivalent to 1 mg/L silver was prepared in ORW and used in the chlorination experiments to account for the effect of silver ions during disinfection.

### Nano-silver particle stability tests

Nano-silver dispersions diluted in various water samples were kept in closed vessels in the dark at 4 °C and at room temperature (24–25 °C) either under continuous magnetic stirring or without stirring for up to 9 months and analyzed every 5 min for the first hour, daily for 1 week, then weekly using a Carry UV 50 Bio spectrometer from Varian Inc. (Palo Alto, CA).

All experiments were conducted at a concentration of 5 mg/L nano-silver particles. An ICP-MS Nexion 300 S (Perkin Elmer, Waltham, MA) was used, when necessary, to verify the presence of silver in solution at concentrations too low to produce a UV absorption spectrum (under 0.1 mg/L).

### Chlorination experiments

ORW and GW samples (see Table 1 for characteristics) containing nano-silver dispersions at 1 and 0.1 mg/L were disinfected by adding sodium hypochlorite solution in sufficient amount to produce a free chlorine residual of 0.25 mg/L after 24 h. Experiments were conducted at the uncorrected pH of the water samples in order to assess the potential catalytic activity of nano-silver.

A subsequent experiment was conducted to account for the effect of nano-silver particles and/or dissolved silver (silver ions). An amount of sodium hypochlorite solution sufficient to produce a 0.4-mg/L free chorine residual after 24 h was added to ORW containing nano-silver, ORW containing an equivalent amount of silver, as AgNO_3_, and ORW without nano-silver to account for the effect of nano-silver particles and/or dissolved silver (silver ions). This experiment was conducted at pH 5, pH 8, and at the uncorrected pH of the ORW. All experiments were conducted in triplicate.

After 24 h, the chlorinated waters were sampled according to the neutral DBP analysis protocol (using ascorbic acid as quenching agent). The samples were then analyzed for 34 neutral DBPs (trihalomethanes, haloacetonitriles, haloacetaldehydes, 1,1 dichloro-2-propanone, 1,1,1 trichloro-2-propanone, chloropicrin, and cyanogen chloride) using a method equivalent to EPA 551.2 that has been described elsewhere (Williams et al. 1997; Kermani et al. 2013).

### Data analysis for the chlorination experiments

Results were compared to the profile of DBPs obtained under the same conditions in the absence of nano-silver and in the presence of an equivalent concentration of Ag+ ions. All experiments were conducted in triplicate. Differences between experiments were compared to differences between replicates.

## Results

### Nano-silver particle stability test results

#### UV absorption spectra of nano-silver dispersions in DW, ORW, and GW

**Fig. 1:**
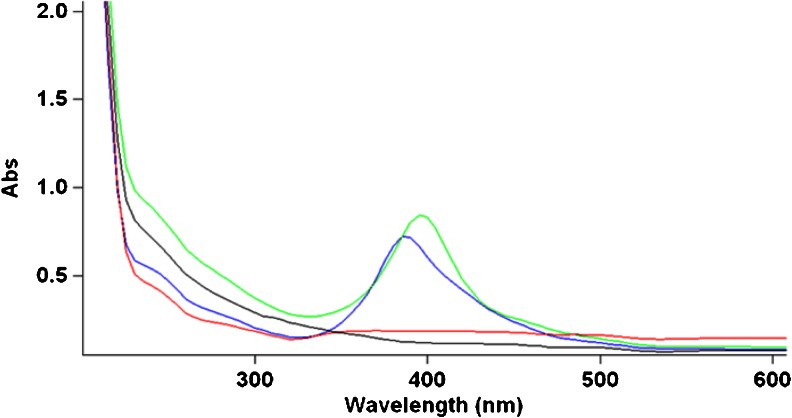
UV spectra of nano-silver dispersion in DW, ORW, and GW after 5 min stirring at room temperature. nAg (10 nm, PVP-coated, 5 mg/L) in DW (*blue*), nAg (10 nm, PVP-coated, 5 mg/L) in ORW (*green*), nAg (10 nm, PVP-coated, 5 mg/L)in GW (*red*), and AgNO_3_ (5 mg/L as Ag+) in ORW (*gray*)

The 10-nm nano-silver particles dispersed in organic-free distilled water present a characteristic UV absorption maximum at 389 nm that is not present in the Ag+ (AgNO_3_) solutions. At only 5 min after the preparation of the 5 mg/L dispersions, the effect of the dispersion media on the stability of nano-silver particles is illustrated in Fig. 1: the absorption maximum of the organic-free, DW dispersion corresponds to a lower nano-silver particles concentration than the one remaining in the ORW (with higher DOC content), while the nano-silver dispersion in the ground water is featureless, corresponding to a nano-silver particle concentration undetectable by UV absorption. Less than 1 % of the initial silver concentration was detected in the soluble fraction in the GW experiment after 5 min.

#### Nano-silver particle stability in DW and ORW under stirred and non-stirred conditions

**Fig. 2:**
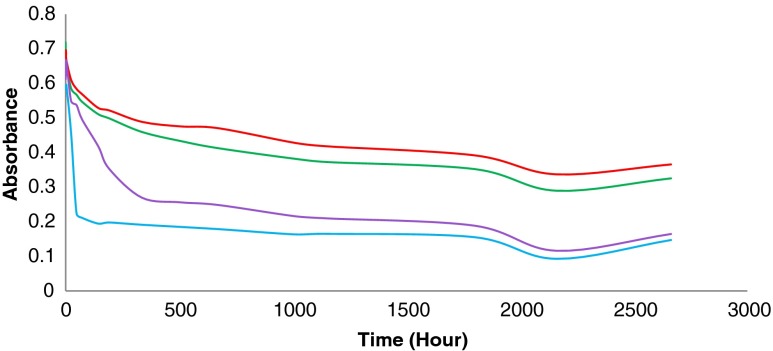
Nano-silver particle stability in DW and ORW under stirred/non-stirred conditions up to 111 days at room temperature. nAg (10 nm, PVP-coated, 5 mg/L) in ORW-non-stirred (*red*) at 395 nm. nAg (10 nm, PVP-coated, 5 mg/L) in ORW-stirred (*green*) at 395 nm. nAg (10 nm, PVP-coated, 5 mg/L) in DW-non-stirred (*purple*) at 389 nm. nAg (10 nm, PVP-coated, 5 mg/L) in DW-stirred (*blue*) at 389 nm

The decrease in concentration of the nano-silver particles in two dispersion media (DW and ORW) with different DOC content was followed by measuring the maximum UV absorption of the dispersions over more than 180 days. Dispersions were kept in the dark at room temperature. Each dispersion was prepared in duplicate, with one of the samples stirred using a magnetic stirrer, while the second one was kept unstirred (Fig. 2).

#### Nano-silver particle stability in treated (chloraminated) drinking water

**Fig. 3:**
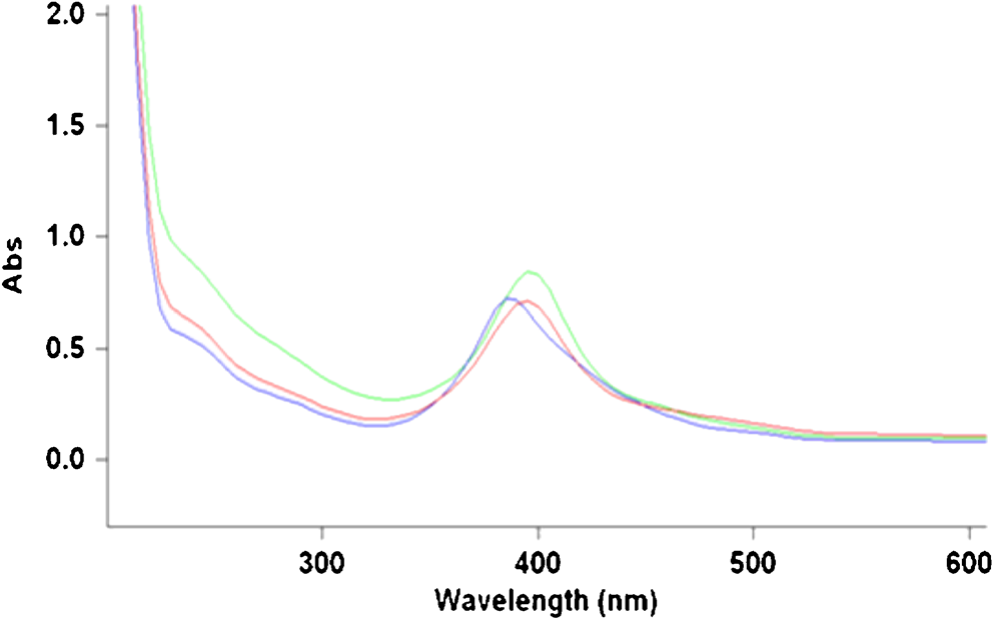
Decrease in the UV absorption maximum of a nano-silver dispersion in OTW after 24 h, at room temperature compared to the absorption maximum of nano-silver dispersions of equivalent concentration in ORW and DW. 5 mg/L of 10 nm nAg, PVP-coated in ORW (*green*); DW (*blue*) and OTW (*red*) after 24 h. OTW has a free chlorine residual of 0 and a total chlorine residual (chloramine) of 1.2 mg/L

**Fig. 4:**
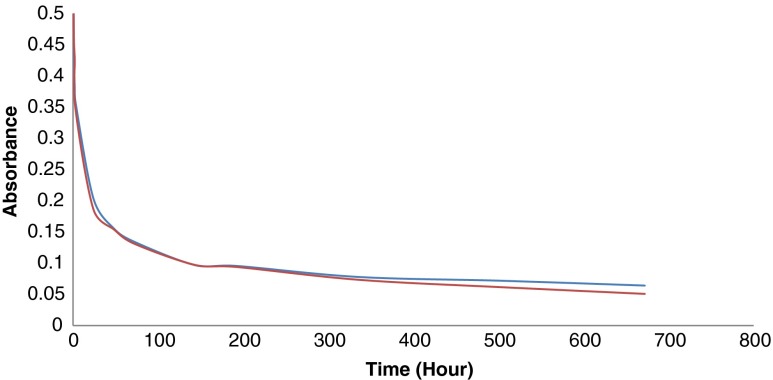
Absorbance at 395 nm of a 5-mg/L dispersion of 10 nm nAg, PVP-coated in OTW non-stirred (*red*) and stirred (*blue*) over 28 days

The stability of nano-silver particles dispersed in OTW, containing chloramines (initial total free chlorine concentration of 1.2 mg/L), kept in the dark at room temperature under stirred/non-stirred conditions for up to 28 days was followed by measuring the UV absorption maximum (Fig. 3 and Fig. 4).

#### Nano-silver particle stability in ORW during chlorination

**Fig. 5:**
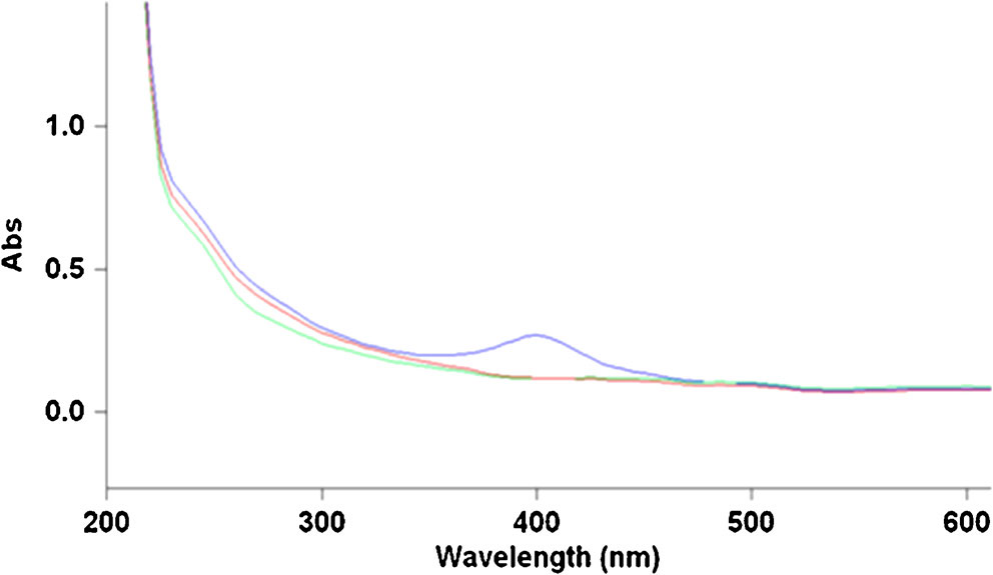
ORW (*red*); ORW with 1 mg/L of 10 nm nAg, PVP-coated (*blue*) and the ORW/nAg dispersion after chlorination (*green*)

The chlorination agent (sodium hypochlorite solution) was added to the dispersion of nano-silver particles in ORW at room temperature. After 5 min contact time (under stirring), a sample of the chlorination mixture was analyzed by UV absorption. The spectrum of the sample was featureless, showing a decrease of nano-silver particle concentration under the detection limit of the method (Fig. 5).

#### Nano-silver particle stability in chlorinated ORW at room temperature over 48 h

**Fig. 6:**
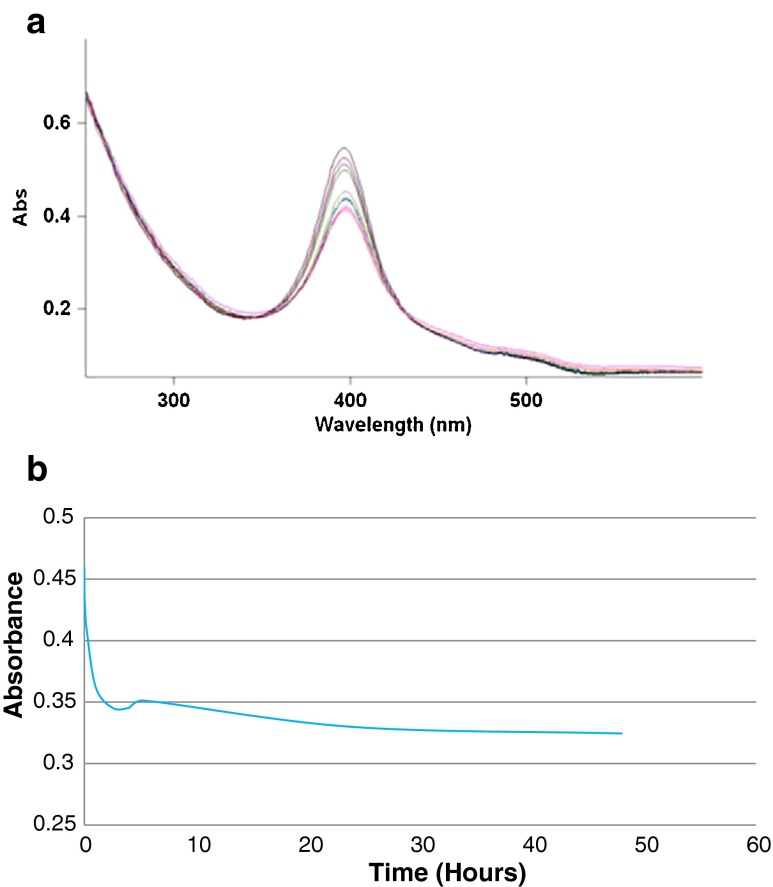
**a** UV absorption of a 5-mg/L dispersion of 10 nm nAg, PVP-coated, added to chlorinated ORW (0.9 mg/L Cl2) over 48 h. **b** Maximum absorption (at 395 nm) change of a 5-mg/L dispersion of 10 nm nAg, PVP-coated, in chlorinated ORW (0.9 mg/L Cl_2_) over 48 h

Nano-silver was added to chlorinated water 24 h after chlorination when the free chlorine concentration was 0.9 mg/L. The rapid decrease in concentration of the nano-silver particles in suspension was followed using UV absorption measurements. Nine samples were measured at uneven intervals in order to capture the quick decrease of UV absorption maximum immediately after addition. Measurements were taken at 1 min, 5 min, 12 min, 1 h, 2.5 h, 4 h, 6 h, 24 h, and 48 h (Fig. 6).

### Results of the chlorination experiments

**Fig. 7:**
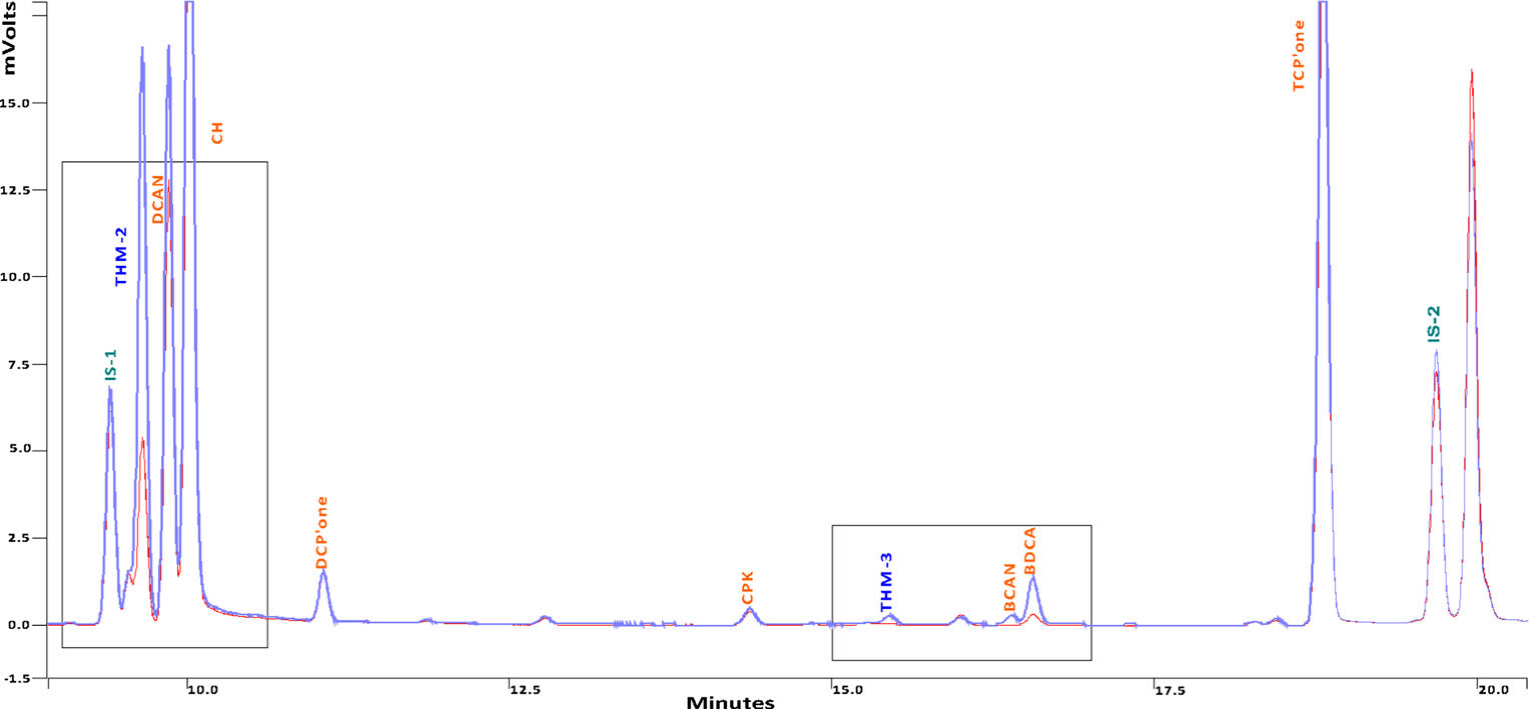
Selection of a chromatogram of neutral DBPs produced by chlorinating 1 mg/L, 10 nm nano-silver, PVP-coated, in ORW (*red*) and by chlorinating the ORW without added nano-silver (*blue*)

DBP concentrations produced in ORW after chlorination in the absence of nano silver, the presence of nano-silver (10 nm, PVP-coated), and the presence of an equivalent amount of silver ions (as AgNO3) are presented in Table 2. Figure 7 shows a comparison between relevant regions of the gas chromatograms of DBPs obtained by the chlorination of ORW in the presence and in the absence of nano-silver (1 mg/L).

**Table 2:** DBP concentrations produced in ORW

	Chlorination, no silver	Chlorination, silver ions	Chlorination, nano-silver
Trihalomethane concentrations—ORW (µg/L)			
Chloroform (TCM)	162.56 ± 12.24	153.03 ± 5.50	153.11 ± 6.53
Bromodichloromethane (BDCM)	8.37 ± 0.26	3.82 ± 0.02	4.24 ± 0.08
Chlorodibromomethane (CDBM)	<0.33	<0.33	<0.33
Bromoform (TBM)	<0.33	<0.33	<0.33
Haloacetaldehydes concentrations—ORW (µg/L)			
Dichloroacetaldehyde (DCA)	0.75 ± 0.05	0.87 ± 0.03	0.81 ± 0.02
Chloral Hydrate (CH)	15.53 ± 0.64	16.45 ± 0.24	15.62 ± 0.63
Bromochloroacetaldehyde (BCA)	<0.02	<0.02	<0.02
Dibromoacetaldehyde (DBA)	<0.03	<0.03	<0.03
Bromodichloroacetaldehyde (BDCA)	0.67 ± 0.04	0.27 ± 0.004	0.28 ± 0.01
Chlorodibromoacetaldehyde (CDBA)	<0.04	<0.04	<0.04
Tribromoacetaldehyde (TBA)	<0.05	<0.05	<0.05
1,1-Dichloro-2-propanone	0.42 ± 0.004	0.60 ± 0.01	0.51 ± 0.01
1,1,1-Trichloro-2-propanone	7.61 ± 0.17	7.61 ± 0.08	5.82 ± 0.13
N-containing compounds concentrations—ORW (µg/L)			
Trichloroacetonitrile (TCAN)	0.05 ± 0.00	0.05 ± 0.00	0.04 ± 0.00
Dichloroacetonitrile (DCAN)	6.40 ± 0.26	6.60 ± 0.12	5.74 ± 0.11
Bromochloroacetonitrile (BCAN)	0.19 ± 0.02	0.08 ± 0.00	0.08 ± 0.00
Dibromoacetonitrile (DBAN)	<0.02	<0.02	<0.02
Chloropricrin (CPK)	0.55 ± 0.01	0.56 ± 0.01	0.56 ± 0.01
Cyanogen chloride (CN-Cl)	0.76 ± 0.07	1.38 ± 0.00	1.03 ± 0.03

## Discussion

### A shift in the absorption maximum

**Fig. 8:**
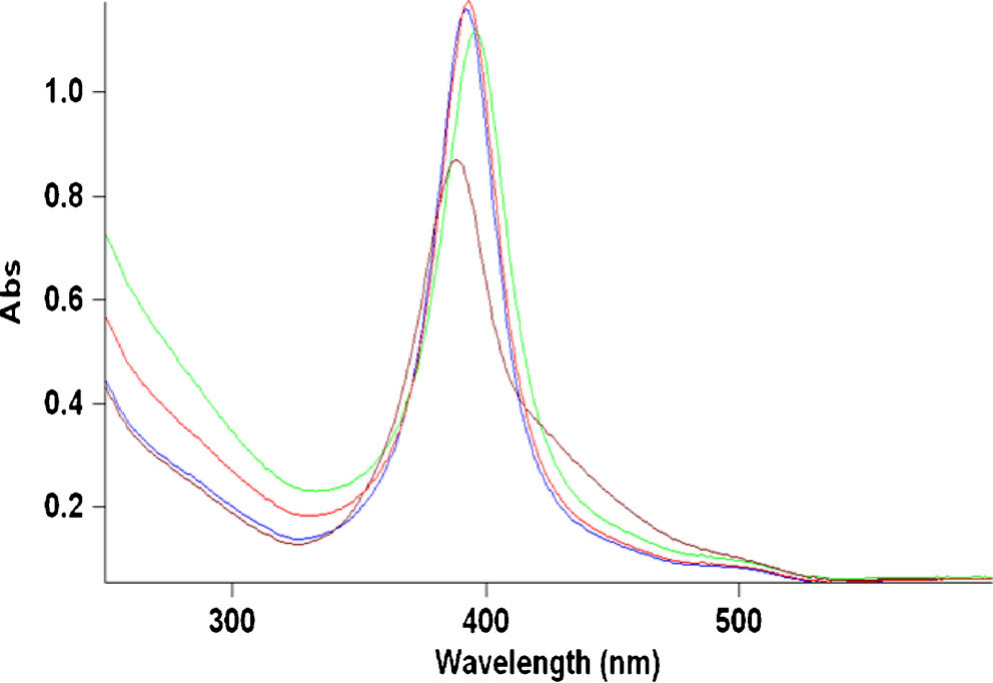
UV absorption curves for solutions of 5 mg/L of 10 nm nAg, PVP-coated in fulvic acid-FA (3.2 mg/L DOC, *red*), FA (0.32 mg/L DOC, *blue*), ORW (6 mg/L DOC, *green*), and DW (*brown*) after 5 min

A slight shift was observed in the absorption maximum (395 nm) of the nano-silver dispersions in ORW (containing 6 mg/L DOC) compared to the absorption maximum (389 nm) of nano-silver dispersions in organic-free distilled water (Fig. 1). The same shift was observed for nano-silver dispersions in OTW (2.6 mg/L DOC, not shown) and in SRFA containing 6 mg/L fulvic acid (3.2 mg/L DOC) and 0.6 mg/L fulvic acid (0.32 mg/L DOC) (see Fig. 8). A slight broadening of the peak was also observed, suggesting changes in size distribution of the nano-silver particles.

A mathematical addition of the ORW absorption curve and the 5 mg/L nano-silver dispersion in DW absorption curve (performed using the Mathlab software) featured an absorption maximum at 389 nm similar to the dispersion in DW. Our hypothesis is that the shift in absorption maximum is due to an interaction of the nano-silver particles with the DOM in the natural water. However, no shift was observed when the nature of the DOM fraction changed (ORW versus OTW and SRFA) or when the concentration of the DOM changed (SRFA at 6 mg/L versus SRFA at 0.6 mg/L). Also, the shift in absorption maximum did not appear to be pH dependent. Adegboyega et al. (2013) observed similar changes in UV absorption curves of nano-silver particles formed by the interaction of Ag+ ions with various humic fractions in solution. In their experiments, the nature of the DOM fraction was shown to influence the features of the UV absorption curves. The difference in results may be explained by the fact that our experiments were conducted at room temperature, where interactions are slower, and we did not allow for sufficient contact time between the nano-silver particles and the DOM fraction solutions.

### Stability of nano-silver dispersions in natural waters

The stability of the nano-silver dispersions in Ottawa River water (with a DOC concentration of 6 mg/L) was significantly higher than the stability of the nano-silver dispersions in distilled, organic-free water (Fig. 1). Nano-silver particles dispersed in groundwater quickly agglomerated and separated from solution (less than 1 % of the silver remained in solution after 0.5 h, as determined by ICP-MS). This was expected based on ion strength effects on nano-silver particles previously described in the literature (Akaighe et al. 2012; Gilliland 2011).

Stirring had a limited effect on the nano-silver particles’ stability, with the effect of stirring being more pronounced in the DW dispersions (Fig. 2).

Our data confirm previous observations that natural dissolved organic matter stabilizes nano-silver particles (Dubasa and Pimpanb 2008; Gilliland 2011) at least in waters with moderate ionic strength and moderate concentrations of divalent ions. The increased stability of the nano-silver dispersions could be explained by nanoparticle adsorption on humic and/or fulvic aggregates in solution or by coating of the nanoparticles with a humic negatively charged layer. In the latter case, the new coating could replace the PVP coating or an “overcoating” of humic could be added. Arguments can be found in the literature for both hypotheses: humic aggregates in solution tend to be a few orders of magnitude larger than the nano-silver particles tested here (Tugulea et al. 2001) and some TEM images appear to show the humic material coating nano-silver particles formed from silver ions in solution containing humic acids (Akaighe et al. 2011). It would be important to establish a definitive mechanism for this process that will allow us to develop a model including the quantitative contribution of DOM concentration, ionic strength, and divalent cation concentrations to nano-silver particle stability in natural waters, such a model would be an important predictive tool.

### Stability of nano-silver dispersions in treated (disinfected) waters

Under oxidizing conditions, Ag is oxidized to Ag+ ions. As expected, nano-silver was not stable during chlorination. The disappearance of the absorption peak at 395 nm, characteristic for the nano-silver particles suspended in the ORW, at 5 min after the addition of the chlorination agent (sodium hypochlorite solution) was observed (Fig. 5). However, nano-silver particles survived for many days when added to the already chlorinated Ottawa River water (with a 0.9 mg/L free chlorine residual) (Fig. 6b) and to the Ottawa River water treated with chloramines (with 0 free chlorine and 1.2 mg/L total chlorine residuals) (Fig. 4).

### Disinfection by-product formation in the presence of nano-silver

The differences observed in the profile of DBPs formed in the presence of 0.1 mg/L nano-silver were in the same range of magnitude as the differences observed between replicates for similar reaction conditions (results not shown).

The profile of DBPs formed in the presence of nAg was significantly different from the profile of DBPs formed in the absence of nAg only for the 1 mg/L nAg concentration (see Fig. 7). The differences observed consisted mainly in the reduced formation of some brominated DBPs (Table 2) and a small increase in the formation of cyanogen chloride (Table 2, N-containing compounds concentrations—ORW). Some of the differences may be explained by the decrease in available bromide due to precipitation in the presence of Ag+ ions (as proven by similar changes observed in the silver nitrate experiment).

The differences observed in the formation of cyanogen chloride appear to be an effect of silver ions in solution: the highest amount of cyanogen chloride was observed for the silver nitrate experiment. Due to the low concentration of cyanogen chloride formed (micrograms per liter), the enhanced formation could be due to the presence in solution of the PVP polymer used as capping agent for the nano-silver product used in this study. The PVP polymer has a relatively high nitrogen content and could be present in the solution in concentrations reaching hundreds of micrograms per liter, potentially providing a good source for the formation of the added amounts of cyanogen chloride. However, PVP was not present in the silver nitrate experiment, which produced the highest amount of cyanogen chloride. With the potential exception of enhanced cyanogen chloride formation, no catalytic effect was observed for 10 nm nAg PVP-coated during chlorination.

## Conclusions

As previously observed, the DOM appears to increase nano-silver particle stability in surface waters. In our experiments, nano-silver was highly unstable in ground water (low DOC, high-ionic strength), with the silver ions precipitating out of the water phase. However, 10 nm nano-silver particles, PVP-coated, were stable in natural surface water for more than a year. A long lifespan of nano-silver particles in surface waters makes it more likely that nano-silver particles will reach drinking water treatment systems. The contributions of DOM concentration, ionic strength, and divalent ions to the stability of nano-silver particles and the interdependence of these factors need to be further studied.

Ten nanometer, PVP-coated, nano-silver appears to be stable in chlorinated drinking water and in chloramine-treated drinking water for more than a week. That raises the possibility that nano-silver, introduced into finished drinking water obtained from a surface water source, may be stable enough to reach the consumers. More research is needed into the potential exposure and types of nano-silver reaching consumers’ taps and the potential exposure to nano-silver which could be released from point of use/point of entry drinking water treatment devices.

The chlorination process will likely destroy nano-silver particles present in natural surface water sources. The 10-nm nano-silver, PVP-coated, at 1 mg/L concentrations has minor effects on DBP formation (stoichiometric reduction of bromine congener formation and increase in cyanogen chloride formation) and they appear to be explained by the effect of Ag+ ions. No catalytic effect was observed. These results need to be verified using a variety of natural surface waters.

It should be noted that the 1-mg/L concentration of nano-silver used in this study is much higher than levels that would be expected to be present in drinking water sources. These results could, however, be significant for the disinfection and subsequent release of some wastewaters with comparably high nAg concentrations into receiving waters.
